# Evidence for sex-specific intramuscular changes associated to physical weakness in adults older than 75 years

**DOI:** 10.1186/s13293-023-00531-w

**Published:** 2023-07-10

**Authors:** Jelle C. B. C. de Jong, Lars Verschuren, Martien P. M. Caspers, Marjanne D. van der Hoek, Feike R. van der Leij, Robert Kleemann, Anita M. van den Hoek, Arie G. Nieuwenhuizen, Jaap Keijer

**Affiliations:** 1 Human and Animal Physiology, Wageningen University, P.O. Box 338, 6700AH Wageningen, The Netherlands; 2 Department of Metabolic Health Research, The Netherlands Organization for Applied Scientific Research (TNO), Leiden, The Netherlands; 3 Department of Microbiology and Systems Biology, The Netherlands Organization for Applied Scientific Research (TNO), Zeist, The Netherlands; 4 Applied Research Centre Food and Dairy, Van Hall Larenstein University of Applied Sciences, Leeuwarden, The Netherlands; 5 MCL Academy, Medical Centre Leeuwarden, Leeuwarden, The Netherlands; 6 Research and Innovation Centre Agri, Food and Life Sciences, Inholland University of Applied Sciences, Delft and Amsterdam, The Netherlands

**Keywords:** Gender, Myofiber type, Frailty, Muscle-aging, Inflammation, Older adults

## Abstract

**Background:**

Physical weakness is a key component of frailty, and is highly prevalent in older adults. While females have a higher prevalence and earlier onset, sex differences in the development of frailty-related physical weakness are hardly studied. Therefore, we investigated the intramuscular changes that differentiate between fit and weak older adults for each sex separately.

**Methods:**

Male (*n* = 28) and female (*n* = 26) older adults (75 + years) were grouped on the basis of their ranks according to three frailty-related physical performance criteria. Muscle biopsies taken from *vastus lateralis* muscle were used for transcriptome and histological examination. Pairwise comparisons were made between the fittest and weakest groups for each sex separately, and potential sex-specific effects were assessed.

**Results:**

Weak females were characterized by a higher expression of inflammatory pathways and infiltration of NOX2-expressing immune cells, concomitant with a higher *VCAM1* expression. Weak males were characterized by a smaller diameter of type 2 (fast) myofibers and lower expression of *PRKN*. In addition, weakness-associated transcriptome changes in the muscle were distinct from aging, suggesting that the pathophysiology of frailty-associated physical weakness does not necessarily depend on aging.

**Conclusions:**

We conclude that physical weakness-associated changes in muscle are sex-specific and recommend that sex differences are taken into account in research on frailty, as these differences may have a large impact on the development of (pharmaceutical) interventions against frailty.

*Trial registration number:* The FITAAL study was registered in the Dutch Trial Register, with registration code NTR6124 on 14-11-2016 (https://trialsearch.who.int/Trial2.aspx?TrialID=NTR6124 ).

**Highlights:**

• In female, but not male older adults, physical weakness was associated with a higher expression of intramuscular markers for inflammation.

• In male, but not female older adults, physical weakness was associated with a smaller diameter of type 2 (fast) myofibers and lower *PRKN* expression.

• Fit older adults (of both sexes) maintained expression levels comparable to young participants of weakness related genes, differing from frail participants.

**Supplementary Information:**

The online version contains supplementary material available at 10.1186/s13293-023-00531-w.

## Introduction

Physical weakness is a key component of frailty, and is highly prevalent in older adults [1, 2]. Frailty greatly decreases quality of life, and is associated with multiple comorbidities, such as chronic obstructive pulmonary disease, diabetes mellitus type 2 and cardiovascular disease, and mortality [3–5]. Importantly, the prevalence of frailty is projected to increase, as the number of older adults will increase world-wide [6]. Therefore, it is pivotal to increase our understanding of the various pathophysiological aspects of frailty and to identify potential therapeutic targets.

Strikingly, frailty is more prevalent in females compared to males at all ages, both in humans [1, 2] and pre-clinical models [7]. The observed sex difference in the prevalence of frailty could potentially be explained by sex-specific biological mechanisms underlying physical weakness. However, studies reporting the results for males and females separately are scarce, as are studies that include matched male and female groups. Moreover, studies that included both male and female groups primarily measured circulating components [8–10]. In a cross-sectional study associations were found between circulating immune cell subpopulations and frailty in females, but not in males [8]. In line with this finding, a longitudinal study showed that circulating C-reactive protein and fibrinogen levels can predict the incidence of frailty in females, but not in males [9], indicating a potential female-specific role of inflammation in frailty. Male specific associations between frailty and circulating vitamin D levels [10] and duration of sleep have also been reported [11]. Together, these studies suggest the existence of sex-specific mechanisms underlying frailty, but studies on intramuscular molecular changes associated with frailty have not been performed for each sex separately, while these could be highly relevant for physical weakness and especially useful for the identification of potential therapeutic targets for physical weakness.

In the FITAAL study, fit and (pre-)frail older adults of both sexes were included, and the male and female groups were matched in regard to age, BMI and the Fried frailty score [12]. Previously, we compared old vs. young groups for each sex separately, to assess sex differences in muscle-aging [13]. Here, we used this dataset and ranked the older adults according to three frailty-related physical performance criteria. Subsequently, we grouped them into ‘fittest’ and ‘weakest’ older adults and investigated the intramuscular parameters that differentiate fit from weak participants, for each sex separately. We set out to find indicators for sex-specific mechanisms in the development of physical weakness.

## Materials and methods

### Study design

Male and female older adults (75 + years of age) were recruited during the FITAAL study [12]. These participants were screened for exclusion criteria including cardiac failure, COPD, anemia, dementia, cancer, neuromuscular disorders, a serious medical event in the past 3 months, enrollment in another study, intake of carnitine supplements or the use of different types of medication. This resulted in the inclusion of 28 male and 26 female older adults, who were matched for age, BMI and the Fried frailty index score. In addition, 13 young male and 13 young female subjects who were matched for age and BMI were recruited as well. The study was approved by the medical ethical committee of Wageningen University (METC nr. 16/20) and is registered in the Dutch Trial Register (NTR6124). All participants provided written informed consent prior to enrollment. An overview of all old and young groups can be found in Additional file 4: Table S1.

### Selection of the weakest and fittest older adults

To identify the fittest and weakest older adults, the older adults were ranked based on their physical function (measured as described below) for each sex separately. A rank for three individual frailty-related physical tests was calculated, and the average of those ranks was used to select the top eight and bottom eight participants for the weakest and fittest group, respectively.

### Body composition and functional tests

Body weight and height were measured and body composition was measured using a DEXA-scan (Hologic Discovery-A, Hologic Inc, Bedford, MA, USA). Physical function was assessed using a five chair stands test, handgrip strength test and a 400-m walk test. These tests were performed as validated previously [14–16]. For the 400-m walk time a time limit of 900 s was used, which was given as a final score if participants were unable to finish the 400-m walk test [14]. For the grip strength, the participants were seated in an upward position with their dominant arm positioned in a 90° angle and the average score of three trials was used [15]. For the five time chair stands test, participants were seated on a chair with arms folded across their chest. The time required to finish the fifth stand was used [16].

### Muscle biopsy and RNA-sequencing

Muscle sampling and RNA-sequencing was done as described previously [13]. Briefly, muscle tissue samples were collected by a trained physician at Leeuwarden medical Centre and taken by percutaneous needle biopsy (50–80 mg) from *musculus vastus lateralis* according to the Bergström method with suction [17, 18]. Samples were taken after an overnight fast, under local anesthesia and taken at the thickest part of the muscle, approximately 15 to 20 cm above the patella. After retrieval, samples used for RNA-sequencing were snap frozen in liquid nitrogen and samples used for immunohistochemistry were snap frozen in isopentane cooled in a liquid nitrogen bath. Subsequently, all samples were stored at − 80 °C. Total RNA was extracted from the muscle biopsies using RNA isolation kit with NucleoSpin columns (Macherey–Nagel, kit#740955). Total RNA concentration was determined spectrophotometrically using Nanodrop 1000 (Isogen Life Science, De Meern, The Netherlands), and RNA quality was assessed using the 2100 Bioanalyzer (Agilent Technologies, Amstelveen, The Netherlands). The NEBNext Ultra Directional RNA Library Prep Kit for Illumina was used to process the samples according to the protocol "NEBNext Ultra Directional RNA Library Prep Kit for Illumina" (NEB #E7420S/L). Strand-specific messenger RNA-sequencing libraries were generated and sequenced at GenomeScan (Leiden, The Netherlands). The libraries were multiplexed, clustered, and sequenced on an Illumina NextSeq500 with a single-read 75-cycle sequencing protocol, 15 million reads per sample. The reference genome and annotation file of Homo_sapiens. GRCh38 was used for analysis in FastA and GTF format. The reads were aligned to the reference sequence and based on the mapped read locations and the gene annotation a read was mapped on the transcript region. These count data were used as input in the statistical analysis using DEseq2 pipeline [19].

### Transcriptome data analysis

RNA-seq-derived count data were normalized and log2fold change values and *p-*values of the weakest (*n* = 8) vs. fittest (*n* = 8) groups and old (all participants, for males *n* = 28 and females *n* = 26) vs. young were calculated for each sex separately using the DESeq2 package in R [19]. Genes were considered significantly differently expressed if p-values were lower than 0.01. For pathway analysis, differentially expressed genes (DEGs) were used as input for Ingenuity Pathway Analysis (www.ingenuity.com, accessed 2022). For upstream regulator analysis, Ingenuity Pathway Analysis software was used as well, in which the activation status of upstream regulators is predicted based on expression changes of known target genes. The heatmaps and principal component analysis plots were created using MetaboAnalyst 5.0 (www.metaboanalyst.ca, accessed 2022). Protein–protein interaction networks were created using DEGs as input in STRING v10 (www.string-db.org, accessed 2022). The gene expression dataset can be accessed from the GEO-database with accession number GSE144304.

### Immunohistochemistry

Sections of 7 µm were cut using a cryostat with temperature set to − 20 °C and sections were stored at − 80 °C until analysis. Sections were air-dried for 30 min and fixated in 4% paraformaldehyde for 15 min. Antigen retrieval was performed by incubating the sections in a sodium citrate buffer (10 mM, pH 6), that was kept at sub-boiling temperature for 15 min using microwave heating. The free aldehyde groups were masked by a 20-min incubation in 1.5% glycine, and the sections were blocked for 30 min in 5% normal goat serum. The sections were incubated overnight at 4 °C with primary antibodies for myosin heavy chain 7 (MYH7, 1:200, SAB4200670, Sigma-Aldrich), dystrophin (1:100, ab85302, Abcam), cytochrome c oxidase subunit 4 (COX4, 1:200, ab16056, Abcam) or NADPH oxidase 2 (NOX2, 1:300, ab80897, Abcam) diluted in 0.05% acetylated bovine serum (900.099, Aurion). Next, the slides were incubated with goat anti-rabbit IgG Alexa Fluor 488 secondary antibody (1:1000, A-11008, ThermoFisher) or goat anti-rabbit IgG Alexa Fluor 594 secondary antibody (1:1000, A-11012, ThermoFisher). Finally, the slides were counterstained using 4′,6-diamidino-2-phenylindole (DAPI) and covered using Fluoromount-g (0100-01, Southernbiotech).

Tile-scans of whole sections were made at 20 × magnification using a fluorescence microscope (Leica DM6B) and a digital camera (DFC365 FX). The scans were made using z-stacks (5 µm interval), and LAS-X software was used for maximum projection processing. Myofiber size was measured in ImageJ using the minimal Feret’s diameter, since this parameter was reported to be least sensitive for the sectioning-angle [20]. The number of myofiber types was counted manually using whole section pictures by a blinded investigator. For the quantification of NOX2-positive cells, at least four regions of interest were blindly selected and used for the measurements. NOX2-positive cells were identified using ImageJ and normalized for area (per 1 mm^2^). Mitochondria (identified by COX4 immunoreactivity) were imaged by scanning myofibers that were cut in longitudinal orientation using a 100 × magnification with oil, as in longitudinal orientation it has been shown that changes in morphology of intermyofibrillar mitochondria can be observed [21]. Mitochondrial networks were analyzed using ImageJ and average mitochondrial branch length was quantified.

### Serum collection and analyses

Blood samples were taken from participants in the morning after an overnight fast and collected using serum separating tubes (BD diagnostics). Serum was stored at − 80 °C until analysis. Leptin and adiponectin concentrations were determined using a commercial ELISA kit (DY398-05 and DY1065-05, respectively, R&D Systems). Assays were performed according to manufacturer instructions.

### Statistical analysis

Statistical differences (of data other than RNA-seq data) between the weakest vs. fittest groups were determined using an unpaired t-test. If data were not normally distributed, then a Mann–Whitney U test was used. If statistical differences between multiple groups were tested, then a two-way ANOVA, follow by a Tukey’s test for post hoc analysis was used. If data were not normally distributed, then a Kruskal–Wallis test followed by Mann–Whitney U test was used. The normality of data was tested using Kolmogorov–Smirnov and Shapiro–Wilk normality test. Correlations between two variables were tested for significance using a regression analysis. A *p*-value of < 0.05 was considered statistically significant and all values are displayed as mean ± SEM. Two-tailed p-values were used and these analyses were performed using GraphPad Prism version 9 (GraphPad Software, CA, USA) or IBM SPSS Statistics 27 (IBM, NY, USA).

## Results

### Participant characteristics of fittest and weakest groups

**Fig. 1:**
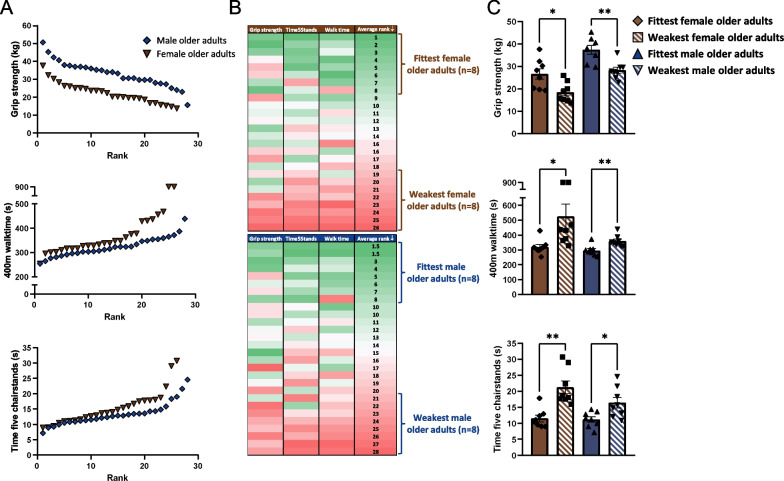
An overview displaying the methodology used to identify the fittest and weakest participants of both sexes. **A** Three graphs displaying the ranking of all male and female older adults based on their grip strength, 400-m walk time and time to perform five chair stands. **B** Two heatmaps displaying the average rank used to identify the fittest and weakest older adults of both sexes. Red or green colors indicate the given rank. A green color corresponds with a higher rank while a red color corresponds with a lower rank **C** Comparisons of the fittest vs. the weakest groups of both sexes separately, based on their grip strength, time to perform five chair stands and time to walk 400-m. All data were normally distributed except time 5 chair stands. Values represent mean ± SEM, *p < 0.05, **p < 0.01

To identify the fittest and weakest older adults, older adults from the FITAAL study were ranked based on their physical performance. Individual ranks were determined for three individual frailty-related physical tests (Fig. 1A), and the average of those ranks was used to select the top eight and bottom eight participants for the fittest and weakest group, respectively (Fig. 1B). This grouping method resulted in significant differences in grip strength (p = 0.01 in females and p = 0.002 in male), 400-m walk time (p = 0.03 in females and p = 0.004 in males) and time to perform five chair stands (p = 0.002 in females and p = 0.02 in males) in the fittest vs. weakest groups of both sexes (Fig. 1C, exact data provided in Table 1). Weight, length and BMI were not significantly different in the fittest vs. weakest groups (Table 1). Age was significantly different in the fittest vs. weakest female groups (*p* = 0.04) and not in the fittest vs. weakest male groups (*p* = 0.14). The relative fat mass was higher in the trunk (*p* = 0.01) and legs of the weakest females (*p* = 0.009), compared to the fittest females. In males, no significant difference was observed in the relative fat mass of the trunk (*p* = 0.34), and a tendency for significant difference was observed in relative fat mass of the legs of the weakest compared to the fittest males (*p* = 0.06). The ratio of fat mass in the trunk vs. legs was not significantly different between the fittest vs. weakest groups of each of the sexes. In line with the observed differences in body composition, average serum leptin concentration was significantly higher in the weakest females compared to the fittest females (*p* = 0.02) and not in the weakest males compared to the fittest males (*p* = 0.17). Adiponectin serum levels tended to be higher in weakest males compared to fittest males (*p* = 0.08), but no such tendency was observed in the female groups (*p* = 0.48).

**Table 1:** Participant characteristics of the fittest and weakest participants for each sex separately

	Females		Males	
	Fittest (*n* = 8)	Weakest (*n* = 8)	Fittest (*n* = 8)	Weakest (*n* = 8)
Handgrip strength (kg)	26.6 ± 2.4^a^	18.5 ± 1.6^b^	37.4 ± 2.1^a^	28.3 ± 1.5^b^
Time 5 chair stands (s)	11.5 ± 1.0^a^	21.2 ± 2.0^b^	11.2 ± 0.7^a^	16.4 ± 1.8^b^
400-m walk time (s)	318.7 ± 17.7^a^	525.3 ± 83.3^b^	295.8 ± 12.0^a^	358.7 ± 14.2^b^
Weight (kg)	70.9 ± 3.5	72.1 ± 3.8	79.4 ± 3.1	81.1 ± 3.7
Length (m)	1.71 ± 0.03	1.71 ± 0.04	1.72 ± 0.04	1.68 ± 0.05
BMI (kg/m.^2^)	25.7 ± 1.3	27.2 ± 1.2	25.4 ± 1.0	27.1 ± 0.9
Age (years)	78.1 ± 0.7^a^	81.2 ± 1.2^b^	79.6 ± 0.6	82.3 ± 1.6
Trunk fat (%)	30.1 ± 1.2^a^	36.7 ± 1.9^b^	25.5 ± 0.7	28.1 ± 1.7
Trunk lean mass (%)	68.5 ± 1.2^a^	62.0 ± 1.8^b^	72.7 ± 0.8	70.5 ± 1.7
Legs fat (%)	37.6 ± 1.1^a^	44.4 ± 1.2^b^	20.7 ± 1.1	25.0 ± 1.5
Legs lean mass (%)	59.1 ± 1.2^a^	52.4 ± 1.0^b^	74.9 ± 1.0	71.1 ± 1.2
Trunk fat (%) / legs fat (%)	0.80 ± 0.03	0.83 ± 0.04	1.24 ± 0.07	1.13 ± 0.04
Leptin (ng/ml)	12.8 ± 2.4^a^	23.1 ± 3.7^b^	6.8 ± 1.3	12.1 ± 3.2
Adiponectin (μg/ml)	5.7 ± 0.8	6.4 ± 0.7	2.0 ± 0.3	3.2 ± 0.5

Letters a-b denote presence of significant difference among fittest vs. weakest groups for each sex separately. Values are averages ± SEM. All data were normally distributed except time 5 chair stands, legs fat and lean mass (%), trunk fat (%) / legs fat (%) and leptin serum concentration

### Diameter of type 2 myofibers is smaller in weak male older adults only

**Fig. 2:**
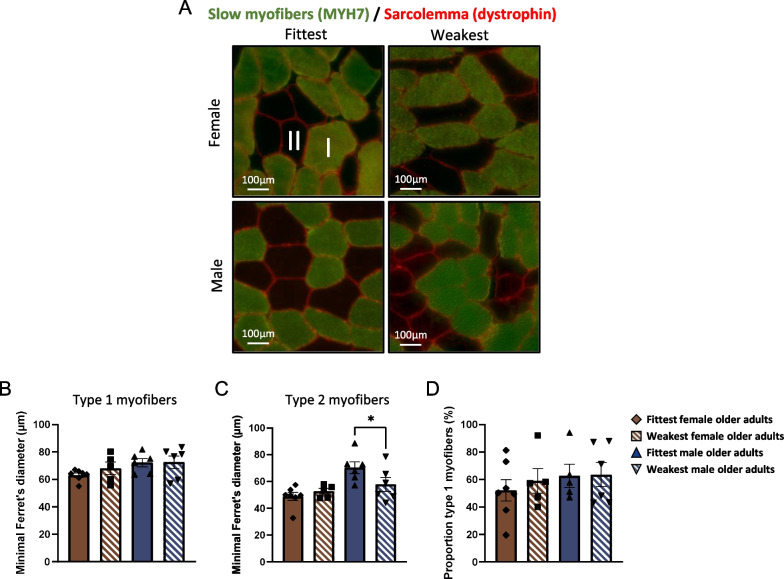
Immunohistological characterization of slow (type 1) and fast (type 2) myofibers. **A** Representative picture of immunohistological staining of slow myofibers (MYH7 in green) and the sarcolemma (dystrophin in red, 20 × magnification). **B** Minimum ferret’s diameter of type 1 and **C** type 2 myofibers. **D** Percentage of type 1 and 2 myofibers. All data were normally distributed. Values represent mean ± SEM, *p < 0.05

The type 1 (slow) and type 2 (fast) myofibers were identified by immunohistochemistry (Fig. 2A). The diameter of the type 1 myofibers was not different in the fittest vs. weakest groups of both sexes (Fig. 2B). The diameter of the type 2 myofibers was bigger in the fittest (70.4 ± 4.4 µm) compared to the weakest (57.9 ± 5.2) male older adults (p = 0.03), but no difference was found between the fittest (52.7 ± 2.1 µm) and weakest (48.9 ± 2.9 µm) female older adults (p = 0.50, Fig. 2C). The proportion of the type 1 myofibers was not different in fittest vs. weakest groups of both sexes (Fig. 2D).

### Physical weakness-associated changes in skeletal muscle transcriptome are sex-specific

**Fig. 3:**
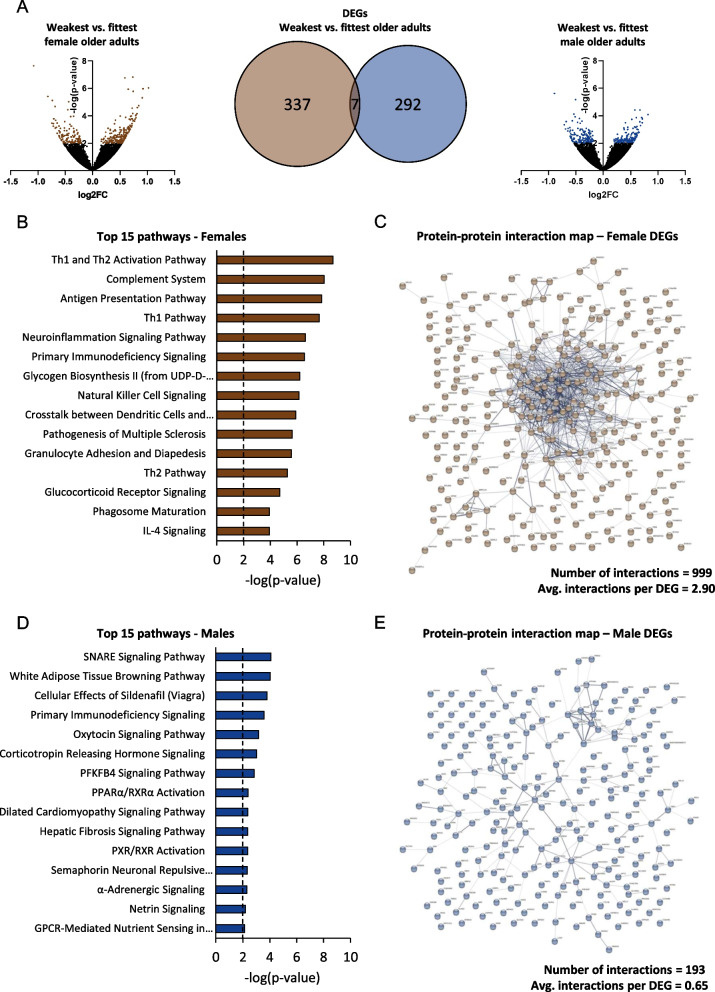
Overview of differentially expressed genes and pathways in *vastus lateralis* muscle RNA-seq data. **A** Volcano plots and Venn diagrams displaying the amount of differentially expressed genes in the weakest vs. fittest older adults (females in brown, left, and males in blue, right). **B** The top 15 differentially expressed pathways in the weakest vs. fittest female older adults. **C** A protein–protein interaction map of all female DEGs. Lines connecting DEGs indicate an interaction between those DEGs on protein level. **D** The top 15 differentially expressed pathways in the weakest vs. fittest male older adults. **E** A protein–protein interaction map of all male DEGs. Lines connecting DEGs indicate an interaction between those DEGs on protein level

To gain insight in the pathways associated to physical weakness, *vastus lateralis* biopsies were used for RNA-sequencing and the weakest vs. fittest groups were compared for each sex separately. In females 344 DEGs and in males 299 DEGs were found (Fig. 3A). Strikingly, only seven of these DEGs were shared between the two sexes, indicating highly sex-specific physical weakness-associated changes in the muscle transcriptome. To gain insight in which biological processes these DEGs are involved, pathway analysis was performed. In females, 45 pathways were significantly differentially expressed and the top ranked pathways were involved in immune cell infiltration and inflammation (e.g., Th1 and Th2 activation pathway, complement system, natural killer cell signaling; Fig. 3B). Additionally, sex-specific PPI networks were built to calculate connectivity of DEGs per sex indicating the relationship between the genes. Protein–protein interaction network mapping confirmed that the identified female DEGs were related, as 999 interactions were revealed between the female DEGs, resulting in on average 2.90 interactions per DEG (Fig. 3C). In males, pathway analysis revealed a much smaller number of pathways, namely 20, which, in contrast to females, were involved in a scattered set of different biological processes, e.g., membrane trafficking (SNARE signaling pathway), energy regulation (white adipose tissue browning pathway) and vasodilation (cellular effects of sildenafil; Fig. 3D). Male protein–protein interaction network mapping revealed 193 interactions, resulting in on average 0.65 interactions per DEG (Fig. 3E), which was much less compared to females. This explains why less pathways were found in the males compared to females, and likely also explains why the male pathways represented a scattered set of biological processes. This was reproduced in the upstream regulator analysis as well, which revealed a predominantly immune-related regulator signature in females, while in the male upstream regulator set such a clear unified signature was not observed (Additional file 1: Fig. S1A, B).

### Female physical weakness is characterized by infiltration of NOX2-expressing immune cells

**Fig. 4:**
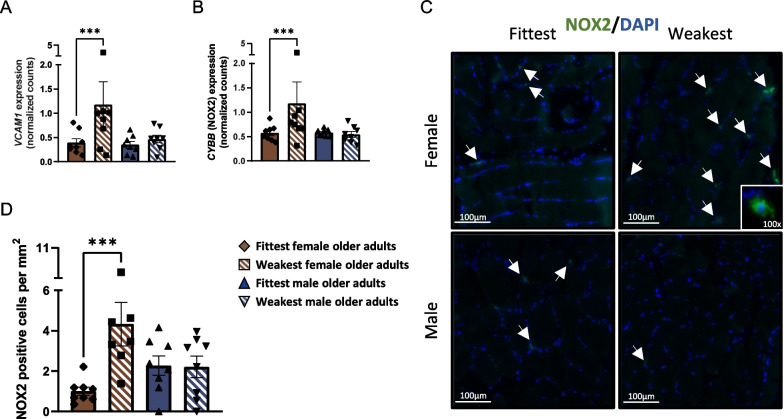
Characterization of female-specific differentially expressed genes and intramuscular immune cells infiltration in the fittest vs. weakest groups of both sexes. **A** Gene expression of *VCAM1* (normalized RNA-seq counts) in the fittest vs. weakest groups of both sexes. **B** Gene expression of *CYBB* (normalized RNA-seq counts), encoding for NOX2, in the fittest vs. weakest groups of both sexes. **C** Representative pictures of NOX2 staining of *vastus lateralis* tissue (20 × magnification). **D** Quantification of immunohistological staining of NOX2, expressed as number of NOX2-positive cells per square millimeter. All data were not normally distributed. Values represent mean ± SEM, ***p < 0.001

Among the top 15 ranked female pathways, key inflammation-related DEGs stood out, such as vascular cell adhesion molecule 1 (*VCAM1*, *p* = 0.0001 in females and *p* = 0.39 in males, Fig. 4A), which mediates adhesion of immune cells and immune cells infiltration [22]. *CYBB* was also one of the top female DEGs (*p* = 0.0002 in females and *p* = 0.56 in males, Fig. 4B), and encodes for NOX2, a subunit of the NADPH oxidase complex that is expressed in inflammatory cells such as macrophages and neutrophils, and mediates production of reactive oxygen species [23]. Together these observations suggest that immune cells infiltration is higher in the weak female group and that those immune cells express NOX2. Indeed, immunohistochemical staining of NOX2 confirmed a greater infiltration of NOX2-expressing immune cells in the weakest (4.3 ± 1.0 NOX2-positive cells per mm^2^) vs. fittest (1.0 ± 0.2 NOX2-positive cells per mm^2^) females (*p* = 0.0009), while this difference was not observed in the weakest (2.2 ± 0.5 NOX2 positive cells per mm^2^) vs. fittest (2.3 ± 0.5 NOX2 positive cells per mm^2^) males (*p* = 0.80, Fig. 4C, D). The number of NOX2 positive cells measured using immunohistochemistry correlated with C*YBB* expression (*r* = 0.60, *p* = 0.02) measured using RNA-seq, thereby cross-validating both techniques (Additional file 2: Fig. S2A).

### Male physical weakness is characterized by a lower parkin expression

**Fig. 5:**
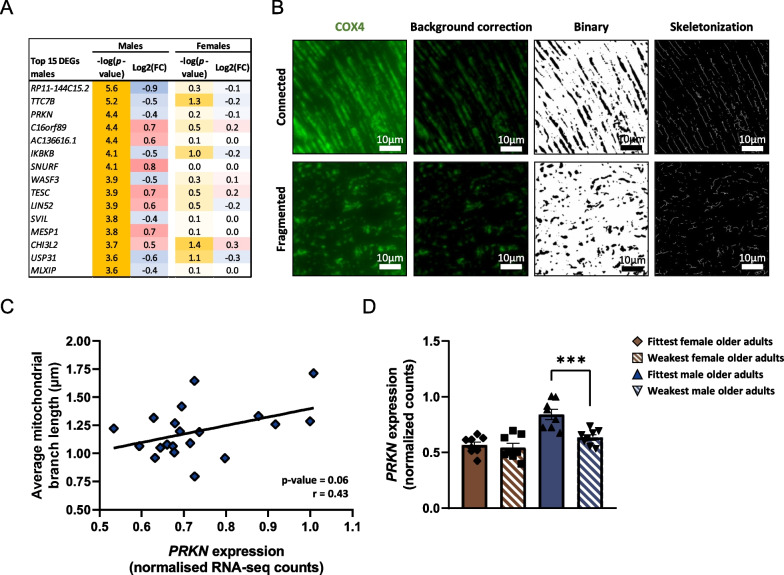
Characterization of top male-specific differentially expressed genes and mitochondrial content in the fittest vs. weakest older adults of both sexes. **A** Top 15 male-specific differentially expressed genes. Blue color indicates a decreased gene expression and red color indicates a higher gene expression in the weakest compared to the fittest groups. **B** Representative pictures of COX4 immunostaining in *vastus lateralis* muscle tissue (100 × magnification). **C** Correlation between the average length of mitochondrial branches and *PRKN* expression (normalized RNA-seq counts) using datapoints from all samples from male older adults with myofibers cut in longitudinal direction (*n* = 20). **D** Gene expression of *PRKN* (normalized RNA-seq counts) in the fittest and weakest groups of both sexes. All data were normally distributed. Values represent mean ± SEM, ***p < 0.01

Since the pathway analysis of male-specific DEGs did not reveal a coherent set of changes in biological processes associated with physical weakness (Fig. 3D, E), we focused on the top DEGs. Among the top 15 male-specific DEGs (Fig. 5A), parkin *(PRKN*) was observed. *PRKN*, which plays a key role in mitophagy and in muscle (mitochondrial) function [24], was significantly downregulated in weakest vs. fittest males (log2FC = − 0.4, *p* = 0.0003). In order to study the effect of the observed lower expression of *PRKN* on mitochondrial function, we stained for COX4 and analyzed the average length of mitochondrial branches as a marker for mitochondrial connectivity (Fig. 5B), which has previously been found to be positively associated with mitochondrial function [25]. Interestingly, *PRKN* expression and average mitochondrial branch length tended to correlate positively (*r* = 0.43, *p* = 0.06, Fig. 5C). No differences in *PRKN* expression were found in the weakest vs. fittest female older adults (*p* = 0.56, Fig. 5D).

### Changes in muscle transcriptome associated to physical weakness are not necessarily a continuum of aging

**Fig. 6:**
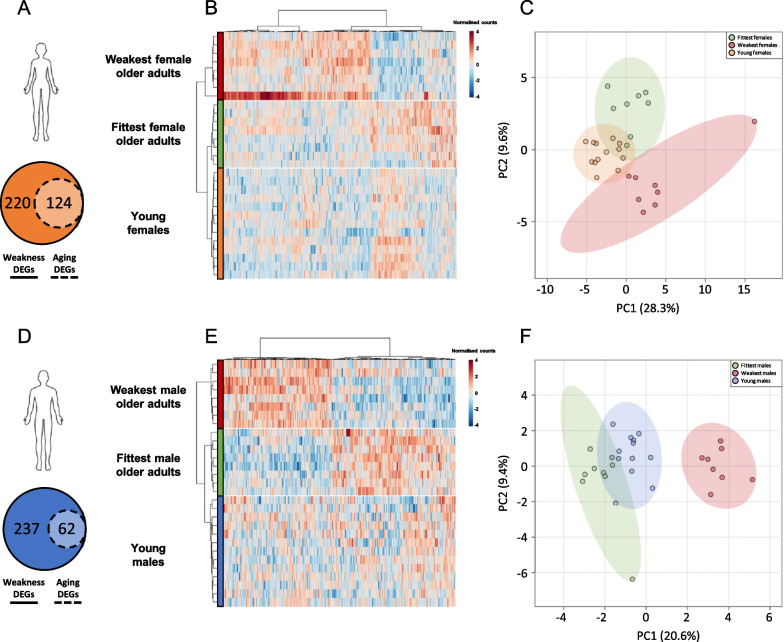
Transcriptional characterization of genes associated to physical weakness, in the context of aging. **A** Venn diagram displaying the number of differentially expressed genes in the weakest vs. fittest female older adults, and the fraction of those differentially expressed genes that were also differentially expressed in old vs. young females. **B** Heatmap displaying the normalized counts of genes that were differentially expressed in the weakest vs. fittest female older adults. **C** Principal component analysis plot based on the 344 differentially expressed genes in the weakest vs. fittest female older adults. Circles around individual data points represent 95% confidence regions per group **D** Venn diagram displaying the number of differentially expressed genes in the weakest vs. fittest male older adults, and the fraction of those differentially expressed genes that were also differentially expressed in old vs. young males. **E** Heatmap displaying the normalized counts of genes that were differentially expressed in the weakest vs. fittest male older adults. **F** Principal component analysis plot based on the 299 differentially expressed genes in the weakest vs. fittest female older adults. Circles around individual data points represent 95% confidence regions per group

Age is a strong predictor of frailty-associated physical weakness, and it could be hypothesized that mechanisms underlying physical weakness are a continuum of aging [26]. To investigate this, we selected the DEGs that discriminated the fittest from the weakest older adults participants, and analyzed whether they were also differentially expressed in a comparison of old vs. young participants. For the latter comparison, not the data from the fittest and weakest older adults were taken, but the data of all older adults were used in the old groups (*n* = 26 for female older adults and *n* = 28 for male older adults) and compared to young participants (*n* = 13 for each sex, see Additional file 4: Table S1 for an overview of all participant characteristics). In females, out of the 344 DEGs that were differentially expressed in the weakest vs. fittest groups, only 124 were also differentially expressed in old vs. young females (Fig. 6A). Normalized expression of each physical weakness DEG was visualized using a heatmap, and the weakest and fittest female older adults and young females were clustered. Notably, the fittest female older adults and young females were clustered together (Fig. 6B), suggesting that in regard to the DEGs associated to physical weakness, the fittest female older adults maintained an expression pattern similar to young females. In agreement, principal component analysis clustered the fittest distinct from weakest female older adults, with the weakest female older adults being slightly further from the young female participants, compared to the fittest female older adults (Fig. 6C). Together, these results suggest that changes in the transcriptome associated to muscle weakness are not a continuum of aging. In males, out of the 299 DEGs that were differentially expressed in the weakest vs. fittest groups, only 62 were also differentially expressed in old vs. young males (Fig. 6D). Normalized expression of each physical weakness DEG was visualized using a heatmap (Fig. 6E), and similar as in the females, the fittest male older adults were clustered together with the young males (Fig. 6E). Principal component analysis revealed an overlap between the young males and the fittest male older adults, whereas the weakest male older adults were separate from the young males and the fittest male older adults (Fig. 6F). This shows that the changes that discriminated the weakest from the fittest older adults did not discriminate old from young participants, and suggests, as was also found in the females, that the changes in the transcriptome associated to muscle weakness in older adults are not necessarily a continuum of aging.

## Discussion

In this study, we investigated for each sex separately which intramuscular alterations are associated with physical weakness. On both the transcriptional and histological level, sex-specific changes were found to be associated with physical weakness. In females, physical weakness was associated with an upregulation of inflammatory pathways. We observed a greater infiltration of NOX2-expressing immune cells on the histological level, concomitant with a greater expression of *VCAM1*. In males, physical weakness was associated with a smaller diameter of type 2 (fast) myofibers and a lower expression of *PRKN*. The expression of *PRKN* also tended to positively correlate with average mitochondrial branch length in males. Lastly, it is generally assumed that mechanisms associated to frailty are a continuum of mechanisms underlying aging. This notion is challenged by our data, as the changes in the transcriptome that were associated with physical weakness were largely not associated with aging.

The most striking finding of this study is that the intramuscular characteristics of physical weakness were highly sex specific, which can have large implications for the development of (pharmaceutical) interventions against frailty. Nevertheless, this finding does not come as a complete surprise. The prevalence of frailty has been reported to be higher in females compared to males, at all ages [1, 2] and also in pre-clinical models for frailty [7]. This suggests that females are either (1) more susceptible to the same weakening mechanisms, or (2) that sex-specific mechanisms underlying physical weakness exist. The latter seems most likely to be true, as previous studies reported a female-specific association between inflammatory blood-based markers and frailty [8, 9]. Here, we expand the paradigm of sex-specific mechanisms underlying frailty, as we now show that intramuscular changes associated with physical weakness are highly sex-specific (at least in *vastus lateralis* muscle). Our data indicated that intramuscular inflammation is associated to physical weakness in females but not in males, which is in line with findings of previous studies that measured associations between circulating inflammatory markers and frailty [8, 9, 27]. Interestingly, another study found that sitting time was associated to blood-based markers of inflammation in females, but not in males [28]. This suggests perhaps that the origin of the sex-specific frailty mechanisms could partly be found in the way males and females respond to physical (in)activity [29, 30]. In future studies accelerometry could be used to measure physical activity as well [31]. Alternatively, loss of inflammatory homeostasis control by estrogens may play a role specifically in (post-menopausal) females [32, 33]. Our findings may have major implications for anti-inflammatory therapies currently being investigated such as TNFα-blocking therapies [34] or COX inhibitors [35].

A frequently mentioned possible explanation for sex differences in frailty is sex differences in body composition. Abdominal adiposity might play a role in inflammation-driven frailty [36, 37]. Trunk fat mass is higher ins females compared to males [38], and consequently it can be hypothesized that trunk fat mass-driven inflammation might play a greater role in frailty in females compared to males. We indeed found a significantly higher relative fat mass in the trunk of the weakest vs. fittest females, but not in the weakest vs. fittest males (Table 1). However, we doubt whether this explains the upregulation of inflammation in the *vastus lateralis* of the weakest females, since relative trunk fat mass did not correlate with the expression of *CYBB*, *VCAM1* or NOX2 (Additional file 3: Fig. S3). More research, in which both males and females are included and compared, is required to investigate the magnitude of the role of trunk fat mass in the sex-specific development of frailty.

The sex-specific mechanisms associated to physical weakness may also be due to different requirements for micronutrients [39]. In a pre-clinical model of vitamin A metabolism evident sex-specific responses were observed in the organs examined [40, 41]. The model consisted of *BCMO1*^*−/−*^ mice, unable to process the provitamin A beta-carotene, but supplied with a sufficient dose of vitamin A. The female mice showed a dysregulation of inflammation related pathways [42]. This was not seen in the male mice which displayed other alterations, especially in development-related processes. This sex-specific response is highly reminiscent of current study, which may possibly suggest that either females have a different immune homeostasis or are more susceptible to, for example, retinoid insufficiency.

In males, completely different biological processes were observed to be associated with physical weakness. Firstly, we observed in males, but not in females, a smaller diameter of type 2 myofibers in the weakest compared to the fittest older adults. In males this has been observed previously [43], but, to the best of our knowledge, a pair-wise comparison as performed here has not been reported before. Aging is known to be associated with a reduction of type 2 myofiber size [44], however, the small age differences of the weakest and fittest groups (∆ 3.1 years in females and ∆ 2.7 years in males) make it rather unlikely that aging could be a confounding factor in our analysis. The size of type 2 myofibers of frail patients can potentially be improved by means of resistance exercise training and protein supplementation, an effect that seems to be mediated by improving myonuclear and satellite cell content [45].

Furthermore, we found that Parkin was positively associated with physical function in males, but not in females. This is surprising, because Parkin plays a key role in mitophagy, and an upregulation of *PRKN* could be associated with an increase in mitochondrial dysfunction or an enhanced removal of damaged mitochondria to improve mitochondrial quality. Our observation, namely a positive correlation between *PRKN* expression, physical function and mitochondrial branch length, point to the latter explanation. This is in line with previous research that showed Parkin is necessary for mitochondrial and muscle function [24], and plays a role in mitophagy following exercise [46]. Perhaps, the observed effect is best explained by a role of Parkin in mitochondrial quality control, enhancing removal of mitochondrial damage, which could be beneficial for aged muscles to improve mitochondrial quality [47], and ultimately muscle function.

Since the prevalence of frailty increases with age, mechanisms that underlie frailty-associated muscle weakness are frequently thought to be a continuum of muscle-aging. We investigated this by comparing whether the transcriptional changes that are associated to physical weakness (weakest vs. fittest older adults) are also associated to aging (all older adults vs. young participants). It became evident after examining the Venn diagrams, heatmaps and principal component analysis plots in Fig. 5, that the changes that discriminate the weakest from the fittest older adults, did not necessarily discriminate old from young participants as well. This second pertinent finding challenges a number of studies, which emphasize the similarities between frailty and muscle-aging, frequently based on the overlap in the phenotypic characteristics of both conditions, such as a decreased grip strength [26, 48]. Here, we show that the underlying mechanisms are not necessarily the same, and that the physical component of frailty deserves to be acknowledged as a pathological process that is potentially independent of, or additional to, muscle-aging. In accordance with this notion, we previously found smaller sex differences in muscle-aging itself [13] compared to the sex differences we found in frailty-associated physical weakness here, which also indicates a lack of overlap between mechanisms underlying muscle-aging and frailty-associated physical weakness.

We conclude that intramuscular features associated to physical weakness are highly sex-specific in older adults. Weak female older adults were characterized by a higher expression of inflammatory pathways and infiltration of NOX2-expressing immune cells, concomitant with an higher *VCAM1* expression. Weak male older adults were characterized by a smaller diameter of type 2 (fast) myofibers and lower expression of *PRKN*. A limitation of the current study is its cross-sectional design, which makes it difficult to take differences in lifestyle into account. In future research, it would be interesting to collect muscle-biopsies over time, to create more insight in the onset and role of the different biological processes that were found to be sex-specific in this study. The novel findings of our study underline that weakness-associated changes in muscle are sex-specific in older adults and we therefore recommend that sex differences are taken into account in research on frailty, as they could have a large impact on the development of (pharmaceutical) interventions against frailty.

## Perspectives and significance

Frailty is hallmarked by physical weakness and is a major public health issue within the older population. The prevalence of frailty has been reported to be higher in females compared to males, but studies investigating potential underlying mechanisms and whether these differ for females and males are lacking. Here, we newly report sex-specific changes associated to frailty-related physical weakness within skeletal muscle tissue. This is a significant finding, as it strongly encourages researchers to take sex differences into account while performing research on frailty-related physical weakness in older adults. In females, but not males, we observed a significant association of physical weakness with a higher expression of intramuscular markers of inflammation, while in males, but not in females, we observed an association of physical weakness with a decreased expression of *PRKN* and size of type 2 (fast) myofibers. These findings were sex-specific and suggest that in order to maximize the beneficial effects of lifestyle or pharmacological interventions, a sex-specific approach might be required. Lastly, we strongly encourage researchers performing (large) longitudinal studies to include both male and female participants and to perform the subsequent analyses for each sex separately, in order to increase our understanding of the role of sex in frailty-related physical weakness.

## Supplementary Information

**Additional file 1: Figure S1.** Upstream regulator analysis displaying the predicted activation state of upstream regulators. **A** The top 20 differentially expressed predicted upstream regulators of the weakest vs. fittest females. **B** The top 20 differentially expressed predicted upstream regulators of the weakest vs. fittest males. A red color corresponds with a predicted increased activation state and a blue color corresponds with a predicted decreased activation state.

**Additional file 2: Figure S2.** Correlation analyses between histological and RNA-seq data of the fittest and weakest older adults of both sexes. **A** Correlation between *CYBB *expression and NOX2 positive cells per mm^2^. **B** Correlation between *MYH2 *expression and the proportion of type 2 myofibers. *CYBB* and NOX2 expression data were not normally distributed. *MYH2* and type 2 myofiber data were normally distributed.

**Additional file 3: Figure S3.** Correlation analyses between relative fat mass in the trunk and inflammatory markers in *vastus lateralis* muscle of the fittest and weakest female older adults. Correlation between *CYBB *expression and relative fat mass in the trunk. Correlation between *VCAM1* expression and relative fat mass in the trunk. Correlation between NOX2 histological signal and relative fat mass in the trunk. All data were not normally distributed.

**Additional file 4: Table S1.** Characteristics of all young and elderly female and male participants of the FITAAL study. Letter a-d denote presence of significant difference among respective groups. Values are averages ± SEM.

## Data Availability

The gene expression dataset can be accessed from the GEO-database with accession number GSE144304. Other data are available from the corresponding author upon reasonable request.

## Abbreviations

COX4: Cytochrome c oxidase subunit 4
CYBB: Cytochrome B-245 Beta Chain
DAPI: 4',6-Diamidino-2-fenylindool
DEGs: Differentially expressed genes
MYH7: Myosin heavy chain 7
NOX2: NADPH oxidase 2
PC: Principal component
PRKN: Parkin
VCAM1: Vascular cell adhesion molecule 1
