# Supplementary material for: Evidence for sex-specific intramuscular changes associated to physical weakness in adults older than 75 years
Source: Biol Sex Differ. 2023 Jul 10;14:45. doi: 10.1186/s13293-023-00531-w (PMC10332038; doi:10.1186/s13293-023-00531-w)
Supplement: Supplementary file 1 — Additional file 1: Figure S1. Upstream regulator analysis displaying the predicted activation state of upstream regulators. A The top 20 differentially expressed predicted upstream regulators of the weakest vs. fittest females. B The top 20 differentially expressed predicted upstream regulators of the weakest vs. fittest males. A red color corresponds with a predicted increased activation state and a blue color corresponds with a predicted decreased activation state. [file 13293_2023_531_MOESM1_ESM.pdf]

Supplementary figure 1

A

Upstream regulator	-log(p-values) Females	-log(p-values) Males	Z-score Females	Z-score Males
IFNG	20.6	0.0	5.9	N/A
IL4	15.5	1.3	1.7	-0.2
Immunoglobulin	14.9	0.6	0.0	2.0
Interferon alpha	13.4	1.6	4.9	0.9
CSF1	12.1	0.0	2.0	N/A
IL2	11.8	0.0	3.7	N/A
Hbb-b2	11.2	0.0	3.1	N/A
CD3	10.8	0.0	1.6	N/A
IL1B	10.6	0.0	3.8	N/A
IL6	10.4	0.3	3.2	-2.2
STAT1	10.0	0.0	4.1	N/A
Hbb-b1	9.9	0.0	2.0	N/A
SLC15A4	9.8	0.0	2.9	N/A
USP22	9.8	0.0	2.7	N/A
STAT3	9.7	0.0	2.1	N/A
TASL	9.4	0.0	2.8	N/A
NFKB1	8.8	0.0	2.8	N/A
FLT3LG	8.8	0.0	2.4	N/A
GRN	8.8	0.0	-0.5	N/A
IFNA2	8.8	0.0	3.5	N/A

B

Upstream regulator	-log(p-values) Females	-log(p-values) Males	Z-score Females	Z-score Males
ALDH2	0.0	3.7	N/A	0.0
Growth hormone	2.5	3.5	0.9	0.8
MESP1	0.0	3.2	N/A	N/A
Fus	0.0	3.1	N/A	-0.6
ENTPD5	0.0	2.9	N/A	N/A
LARP1	0.0	2.7	N/A	2.2
PSMD10	0.0	2.6	N/A	N/A
NOX4	1.5	2.5	N/A	0.4
miR-27a-3p	0.0	2.5	N/A	1.1
APLN	0.0	2.5	N/A	2.0
HCFC1	0.0	2.5	N/A	N/A
SOD1	1.7	2.5	N/A	N/A
NR4A1	2.5	2.3	-0.5	-0.8
PRPF19	0.0	2.2	N/A	N/A
RXRA	1.8	2.2	0.1	2.0
SOX1	0.0	2.1	N/A	1.0
EHMT1	0.0	2.1	N/A	-0.4
GPRC6A	0.0	2.0	N/A	N/A
miR-196a-5p	0.0	2.0	N/A	N/A
PEMT	0.0	2.0	N/A	N/A
