# Supplementary figures and images for: Evidence for sex-specific intramuscular changes associated to physical weakness in adults older than 75 years
Source: Biol Sex Differ. 2023 Jul 10;14:45. doi: 10.1186/s13293-023-00531-w (PMC10332038; doi:10.1186/s13293-023-00531-w)

Supplementary figure 2

A

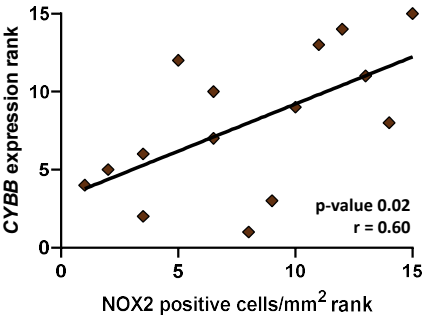

Female older adults  
Male older adults

B

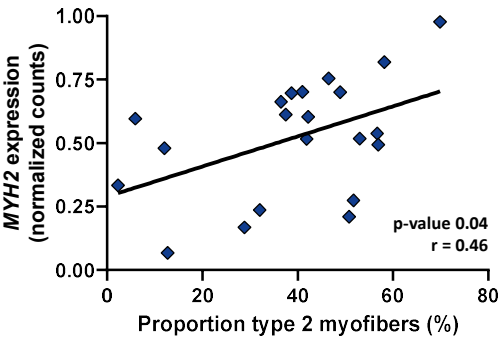

Supplement: Supplementary file 2 — Additional file 2: Figure S2. Correlation analyses between histological and RNA-seq data of the fittest and weakest older adults of both sexes. A Correlation between CYBB expression and NOX2 positive cells per mm2. B Correlation between MYH2 expression and the proportion of type 2 myofibers. CYBB and NOX2 expression data were not normally distributed. MYH2 and type 2 myofiber data were normally distributed. [file 13293_2023_531_MOESM2_ESM.pdf]

Supplementary figure 3

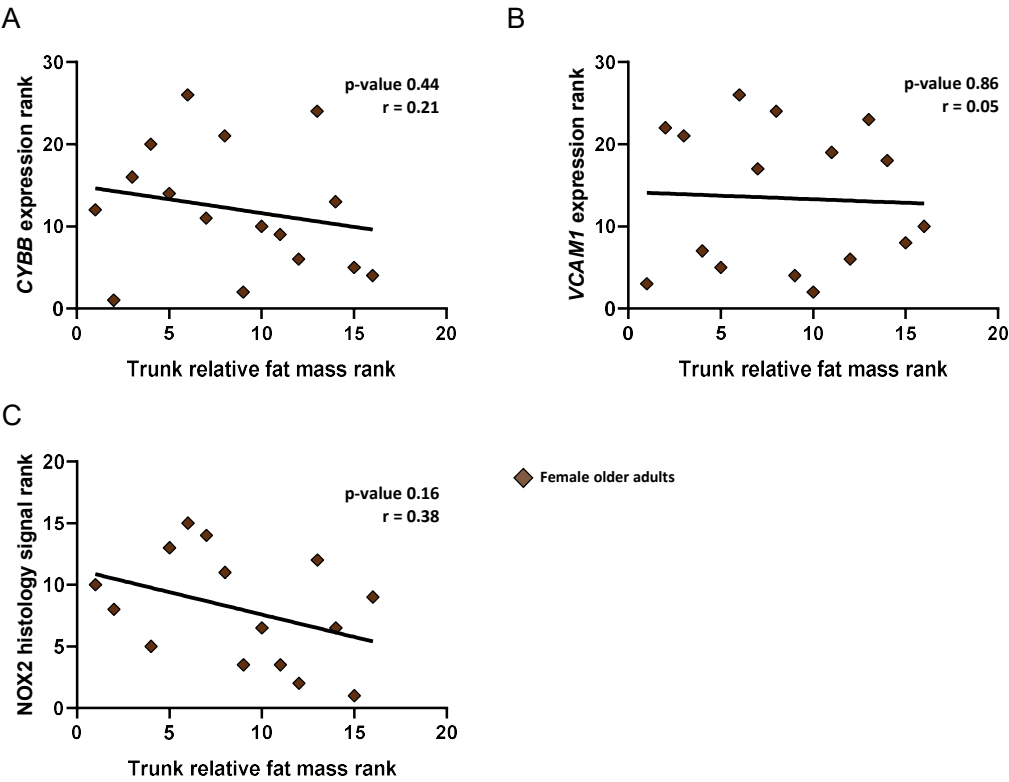

Supplement: Supplementary file 3 — Additional file 3: Figure S3. Correlation analyses between relative fat mass in the trunk and inflammatory markers in vastus lateralis muscle of the fittest and weakest female older adults. Correlation between CYBB expression and relative fat mass in the trunk. Correlation between VCAM1 expression and relative fat mass in the trunk. Correlation between NOX2 histological signal and relative fat mass in the trunk. All data were not normally distributed. [file 13293_2023_531_MOESM3_ESM.pdf]
