# Supplementary material for: Evidence for sex-specific intramuscular changes associated to physical weakness in adults older than 75 years
Source: Biol Sex Differ. 2023 Jul 10;14:45. doi: 10.1186/s13293-023-00531-w (PMC10332038; doi:10.1186/s13293-023-00531-w)
Supplement: Supplementary file 4 — Additional file 4: Table S1. Characteristics of all young and elderly female and male participants of the FITAAL study. Letter a-d denote presence of significant difference among respective groups. Values are averages ± SEM. [file 13293_2023_531_MOESM4_ESM.docx]

**Additional file 4: Table S1:** characteristics of all young and elderly female and male participants of the FITAAL study. Letter a-d denote presence of significant difference among respective groups. Values are averages ± SEM.

|  | Females |  | Males |  |
| --- | --- | --- | --- | --- |
|  | Young (n=13) | Old (n=26) | Young (n=13) | Old (n=28) |
| Age (years) | 22.6 ± 0.55^a^ | 80.2 ± 0.6^b^ | 23.3 ± 0.5^a^ | 79.7 ± 0.7^b^ |
| Weight (kg) | 63.9 ± 1.7^a^ | 68.9 ± 2.0^a^ | 76.2 ± 2.5^b^ | 81.0 ± 2.1^b^ |
| BMI (kg/m^2^) | 22.2 ± 0.6^a^ | 26.2 ± 0.5^b^ | 22.5 ± 0.3^a^ | 26.4 ± 0.7^b^ |
| Body fat (%) | 29.1 ± 1.1^a^ | 35.7 ± 0.8^b^ | 16.9 ± 0.9^c^ | 25.3 ± 0.9^d^ |
| Lean mass (%) | 67.3 ± 1.0^a^ | 61.4 ± 0.8^b^ | 79.3 ± 0.9^c^ | 71.3 ± 0.8^d^ |
| BMC (%) | 3.6 ± 0.1^a^ | 2.9 ± 0.1^b^ | 3.7 ± 0.1^a^ | 3.3 ± 0.1^b^ |
| Handgrip strength (kg) | - | 22.2 ± 1.1^a^ | - | 32.0 ± 1.4^b^ |
| 400m Walk Test (s) | - | 393.9 ± 31.0^a^ | - | 323.3 ± 7.6^b^ |
| Time 5 chairstands (s) | - | 15.2 ± 1.1^a^ | - | 13.3 ± 0.7^a^ |
| Fried frailty score | - | 0.7 ± 0.2^a^ | - | 0.5 ± 0.1^a^ |
| (pre-)frail (%) | - | 42.3%^a^ | **-** | 46.4%^a^ |
